# Perceptions of COVID-19 patients in the use of bioethical principles and the physician-patient relationship: a qualitative approach

**DOI:** 10.1186/s12910-024-01009-z

**Published:** 2024-02-09

**Authors:** Guillermo Cantú Quintanilla, Irma Eloisa Gómez-Guerrero, Nuria Aguiñaga-Chiñas, Mariana López Cervantes, Ignacio David Jaramillo Flores, Pedro Alonso Slon Rodríguez, Carlos Francisco Bravo Vargas, America Arroyo-Valerio, María del Carmen García-Higuera

**Affiliations:** 1 Universidad Panamericana, Mexico City, Mexico; 2 Hospital General de México Dr. Eduardo Liceaga, Mexico City, Mexico

**Keywords:** Physician–patient relationship, Bioethics, Ethics of virtue, COVID-19, In-depth-interviews, Patients-perception

## Abstract

**Background:**

The COVID-19 pandemic has influenced the approach to the health-disease system, raising the question about the principles of bioethics present in physician–patient relations. The principles while widely accepted may not be sufficient for a comprehensive ethical analysis. Therefore, the aim of this study was to explore the perception of these principles and the physician–patient relationship during a hospital stay through a qualitative approach.

**Method:**

Sixteen semi-structured interviews took place to know the patients’ perception during their 2020 hospitalization for COVID-19. The data was analyzed through the constant comparison method, creating categories and comparing them. In the end, seven categories were established and were grouped in three: bioethical principles (dignity, charity, vulnerability, autonomy), doctor-patient relationship (participant commitment, informed consent, health staff-patient relationship) and the experience of the disease (illness, the role of the family).

**Results:**

The research found that most patients described a positive experience, with the feeling of having been well cared for with no sense of discrimination or injustice done. The majority also reported that their autonomy was respected in the treatment decisions. The evaluation of these attitudes is an area of opportunity, especially when the patients' vulnerability is at risk.

**Conclusions:**

The ethics of virtue offers a better reflection of how human beings manifest themselves by emphasizing the development of virtuous character and behaviors that allow them to realize their values in life. Authorized by the Research Ethics Committee with registration: DI/18/105-B/3/308.

**Supplementary Information:**

The online version contains supplementary material available at 10.1186/s12910-024-01009-z.

## Background

The COVID-19 pandemic affected the health-disease conception and the doctor-patient relationship, jeopardizing trust and disrupting traditional healthcare practices. On top of that work overload was accentuated and the lack of resources increased. In addition, the use of protective equipment hindered communication with the patient. Therefore, understanding and kindness towards the patient should become vital [1]. Being admitted to a hospital, having a diagnosis of COVID-19 and being isolated, places the patient in a condition of fragility, impotence and limitation. Consequently, their entire existence, their values ​​and principles of justice, freedom, autonomy, quality of life and dignity become vulnerable [2].

The medical profession, driven by humanistic values, should require practitioners to embody virtues such as fidelity, benevolence, compassion, and justice. In this context, bioethics plays a vital role in humanizing healthcare services by ensuring patient well-being, dignity, rights, and quality of life. This needs a broader approach to healthcare that focuses on holistic healing, respecting patients' rights, autonomy, beliefs, and ultimately safeguarding their dignity and life quality. The doctor-patient relationship was no exception, triggering imbalance in the possibilities of providing health care as used to, putting that relationship of trust at risk [3]. The sick person should be the focus and therefore the treatment needs to be holistic.

Humans are inherently dialogical beings, driven by a desire to understand and seek the best possible care for severe illnesses like COVID-19. The pursuit of virtue is crucial for personal growth, social cohesion, and community well-being. In healthcare, the principles of bioethics, such as dignity, integrity, vulnerability, justice, beneficence, and autonomy, can be viewed as a system of virtues. These principles guide healthcare professionals in providing ethical and compassionate care to their patients, emphasizing qualities like honesty, courage, compassion, and justice [4].

During the COVID-19 pandemic, the instrument ReMePaB was administered to patients admitted to a COVID hospital, addressing their treatment and concerns amidst challenging circumstances. This questionnaire, beyond the Anglo-Saxon principles, considers the Barcelona Declaration and Peter Kemp's proposal, which emphasize patients' dignity, vulnerability, and integrity. The instrument was first applied in the Nephrology service, providing valuable insights to improve the quality of care in bioethical principles [5].

The principles of bioethics considered in the ReMePaB interview are described next. The concept of autonomy emphasizes respecting an individual's right to be recognized, allowing them to express their views, make choices, and act based on personal values and beliefs. Health professionals must encourage voluntary participation and involve patients in decision-making processes [6]. Beneficence involves promoting the well-being of patients and minimizing harm to the greatest extent possible. It encompasses acts of generosity, kindness, benevolence, altruism, love, and humanity. In medical practice, a balance between benefits and risks is necessary [6]. Justice pertains to the fair and reasonable allocation and distribution of limited healthcare resources, especially in the context of a pandemic [6]. In a medical context, vulnerability refers to recognizing and addressing individuals' weaknesses and potential health-related issues. It emphasizes understanding and valuing the fragility of human nature [2]. Dignity refers to the inherent and unconditional value of each person. It entails treating individuals with respect, regardless of physical characteristics, and recognizing their individuality. Preserving dignity, even in illness, is of utmost importance, as it relates to a person's sense of self and vulnerability [7].

This application served both patients and medical personnel, considering the fear of contagion and the care needed for seriously ill patients. The results of this application were published [8]. Following this, a qualitative study was proposed to the hospital bioethics committee, aiming to explore the expanded principles of bioethics and the doctor-patient relationship.

With the approval of the research ethics committee, the proposal was to conduct in-depth interviews to gain insights from the patients' experiences. The study focuses on exploring patients' perceptions of the principles of bioethics and the physician–patient relationship throughout their hospital stay until discharge, complementing the previous quantitative study conducted in the same hospital [8].

## Method

Based on the previously described quantitative approach study [8], this qualitative approach study was designed to explore the perception of patients about the principles of bioethics and the doctor-patient relationship during their hospitalization and at their discharge and was authorized by the Research Ethics Committee with registration: DI/18/105-B/3/308. For this study, we selected the basic qualitative approach described by Merriam [9], which seeks to understand a phenomenon from the participants’ perspective.

The aim of the research was to explore the patients’ perception regarding the presence of the principles of bioethics in the care received during their hospitalization for COVID-19, in the year 2020. The study was guided by the following research questions:

- What is the patient’s perception about bioethical principles during their hospitalization?
- What is the experience of patients hospitalized for COVID-19 in the pneumology unit and later discharged regarding the doctor-patient relationship?
- How did the patients perceive the course of their disease?

### Participants

Sixteen interviews were conducted between January and June 2022 with patients who were hospitalized during 2020 in the context of the COVID-19 pandemic in Mexico City. The interviewees were 5 women, ages 34 to 56 (M = 45 years), and 11 men, ages 29 to 85 (M = 52 years), all from different parts of the country. None of the patients were intubated during their hospital stay.

### Information collection

Convenience sampling was used in which participants from a previous study with COVID-19 patients were contacted to invite them to participate in this new study [8]. A phone call was made explaining the study and inviting them to participate. It was explained to them that this phase consisted of conducting in-depth interviews, and if they agreed to participate, they were scheduled for a call on a different day to conduct the interviews. Prior to starting the interview, the study's purpose was explained, confidentiality was ensured, and permission to record the conversation was obtained.

As the research approach is qualitative, data collection was conducted by one of the study researchers. The in-depth interview technique was employed, using a guide consisting of 27 questions that explored the patients' experiences related to the principles of bioethics and the doctor-patient relationship. The semi-structured nature of the interview allowed for flexibility while supporting a coherent conversation. The interviews were conducted via telephone, with an average duration of 40 min. They were recorded and later transcribed for analysis.

### Information analysis

The qualitative analysis employed in this study is both inductive and comparative. The constant comparison method was used, which involves finding units of information, initially categorizing them, and comparing them to uncover similarities and recurring patterns. Through this process, relationships between categories are established, leading to the determination of final categories [10, 11].

Upon conducting the first interview, it was transcribed for analysis. Four researchers took part in categorizing the interview by finding meaning units and assigning them initial categories. These categories serve as abstractions of the collected data, aiding in its description and interpretation. Each interview was meticulously coded, considering each unit as the smallest meaningful piece of information.

Following the coding and analysis of the first interview, researchers examined the gathered information and adjusted the interview guide, considering the research objectives. Subsequently, a second patient was interviewed, and the process was repeated with a total of 16 participants. The final interview guide was made up of 27 questions organized into six sections (Additional file 1: Appendix 1). Ultimately, seven categories were developed, which were divided into three levels: the first level encompassed bioethical principles, the second level pertained to the doctor-patient relationship, and the final level related to the experience of the disease.

### Validity and reliability

Merriam & Tisdell [12] state that the research must produce "reliable" results, which can be achieved by paying special attention to the conceptualization of the research work, the collection and analysis of information, and the presentation of results. Similarly, Kriukow [13] explains that the most important concept of validity in a qualitative study is reliability. This implies that the findings stand for participants’ perspective. Among the strategies to increase this credibility, also referred to as integrity, the authors propose the researchers' commitment to the study. Four researchers initially participated in this project in the analysis of the information. Three of them have experience in bioethics and took part in the first phase of this study. The interviews were conducted and analyzed weekly. The 4 researchers analyzed the information and critically discussed the findings. As of interview no. 8, 3 sixth-semester medical students were included, who also reviewed the relevant literature for the construction of the theoretical framework.

In addition to the involvement of the researchers, the triangulation supports the validity of the study. As already explained, in a first phase, a quantitative study was conducted whose results make up a form of methodological triangulation that supports the findings of this study. Finally, the authors propose that it is important to keep the transcripts of the interviews and the importance of conducting detailed coding for future reference.

## Results

From the sixteen interviews, 5 to women and 11 to men, all with a COVID-19 diagnostics but none intubated, seven data categories were distinguished. The first and broadest refers to the participants’ experiences on the principles of bioethics, which include dignity, beneficence, vulnerability, and autonomy. The second level includes a category associated with the doctor-patient relationship. The third level refers to the patients' experience of their disease and the role of family.

### Dignity

Patients perceive the concept of dignity from two perspectives, involving both the intrinsic value of individuals and respect towards others. Regarding intrinsic value, participants emphasized that "[…] each person is important and valuable". They linked dignity to self-respect and holistic self-care, emphasizing the need to uphold personal values. Patient 11 mentions that “dignity is […] not to lose values, respect above all for oneself, to take care of the person in an integral way”.

Furthermore, patients associated dignity with respect for others, influenced by the upbringing and education they received. They viewed dignity as having education, being respectful, avoiding envy, and refraining from comparing oneself to others. They acknowledged the role of parental guidance and values in shaping their understanding of dignity: “[…] the education that our parents gave us, the values”. Additionally, gratitude was connected to dignity, with patients considering humility, kindness, and gratitude as integral aspects of it.

Participants also shared instances they perceived as undignified treatment, which involved a lack of respect. Patient 6 expressed that disregarding someone's dignity equates to “ignoring him, belittling him […] not having values ​​towards our fellow men”.

### Charity

Beneficence, as a positive interaction between doctors and patients, is characterized by trust, empathy, and effective communication. Within the category of beneficence, we named four subcategories: justice, solidarity, diligence, and gratitude.

Patients expressed their feeling of receiving help from the care provided by healthcare professionals and receiving fair treatment across all subcategories. They acknowledged the comprehensive support received from doctors, stating that "[…] nothing was missing, and all the doctors helped a lot". Establishing a positive relationship with healthcare personnel was emphasized by many patients, who valued the doctors' attentive listening and considered it instrumental in their rapid recovery and the restoration of hope. The care received was described as good, extensive, sufficient, fast, prompt, and even the best for their COVID-19 recovery journey. Patients appreciated the continuous availability of friendly healthcare providers, showing an important level of attention and diligence: “everyone was very friendly: morning, afternoon and night, yes, without any carelessness”.

In terms of justice, patients reported receiving all necessary services and medications for their treatment. One patient described their experience of being admitted: "I spent about four hours downstairs to be given access to be admitted […] After, I had very good care, they gave me serum, antibiotics, medications”.

In addition to justice, gratitude was constantly reported; possibly, the most frequently mentioned. Some reported feeling grateful during hospitalization: "I had everything at hand, I even felt sorry for the nurses, so much inconvenience and I even said: I think I'm going to stay here for life, hotel and food on order". While others spoke of feeling grateful for having left the hospital: "when I left there, I was very grateful to everyone, with all the nurses, even the cleaner… oh, you can't imagine how grateful I was to them”.

Diligence was also present in the perception of the interviewed patients, finding answers such as: “this… Am I not bothering you too much? [asking the nurse] ―No, no, no Sir, that's my job and we have to be on the lookout― and if at any time I said: I'm going to pee, ―Do you want me to help you down, Don?”; In general, the patients reported feeling always cared for by the staff since they not only covered the medical needs, but also felt accompanied: “yes, there is a nurse who was the first one who helped me walk to the bathroom and waited until I was ready and helped me to bathe and to go to bed”. The patients also recognized the health personnel’s professionalism: “[…] they were always err, well, vigilant, they checked the studies or the X-rays continuously and all that. They also checked everyone, and they did it on time”.

The patients acknowledged the solidarity shown by the healthcare personnel, highlighting their attitudes and actions that made them feel accompanied during their hospitalization. One patient expressed a sense of closeness and kindness from the staff: “Look, I felt very close to them, very kind; morning, afternoon and night, without no oversight”. Patient 12 stated: “He talked with us, and it was a talk about his own experience, life, what he did, and he was very kind to us. He even tried to be very aware of us. He was very, very kind.”. Patient 6 mentioned a significant detail that the staff implemented, which involved providing tablets for patients' relatives to upload videos. Then “a doctor would come up with a tablet and from there he would focus on us, and the person was talking to us about the videos, so I want to think that [it was] a great stimulus for us, patients”.

Interestingly, solidarity among the patients themselves was also observed. They learned to support and care for each other, understanding that they were not the only ones requiring attention. Patient 3 mentioned the importance of recognizing that there were other patients and being understanding when their temperature checks or treatments were delayed due to attending to other individuals. Patient 6 further explained that, later on, they were informed that the video-sharing service was restricted to patients who were unlikely to be discharged, “so they are giving priority to those who were no longer going to be discharge and see their relatives, so they gave them the video to say goodbye to them”.

### Vulnerability

The vulnerability category encompasses three dimensions: fear, dependency, and sadness. Patients expressed fear due to past experiences of losing loved ones to COVID-19, creating concerns about their own fate: “They were in confinement, or they were cremated and thus delivered with their coffin. Not one of them said goodbye. But no, it can't be that this is going to happen to me”. They also felt pressure and anxiety when surrounded by other patients who were in more critical conditions, uncertain of their own prognosis. Additionally, they feared the impact their health status would have on their family life, particularly regarding their ability to support and be present for their loved ones. A patient said “I have a little girl. I mean, finally my wife knows how to work and well, I could have supported her [,] but there is no way to be at home with them […]”.

The dependency dimension highlighted patients' feelings of relying on others and experiencing a partial loss of autonomy during hospitalization. They described a sense of disorientation, not being able to differentiate between day and night, potentially due to medication or the care received: “when the nurses arrived at 10:00 p.m. at night, I always thought it was dawn”. The third dimension, sadness, was expressed in relation to the emotions associated with hospitalization and isolation. Patients described feeling scared, sad, and desperate. However, despite their sadness, they also acknowledged moments of joy and gratitude toward the healthcare personnel for their medical attention. The patients’ improvement conditions gave rise to relaxation and humor: “I joked with the doctors, I told them that after the liposuction they did to me, I no longer have buttocks or boobs”.

### Autonomy

Autonomy forces health personnel to explore the patient's will and enhance their decision-making [6]. In this study it is presented in two dimensions. The first refers to the commitment and responsibility that the participants experience when contributing to their treatment, both inside and outside the hospital, as well as the desire to move forward. The second refers to informed consent as a "tangible expression of respect for people’s autonomy, which includes the right to information and freedom of choice" [14].

#### Participant commitment

Autonomy appeared as a prominent motivational factor among the participants, emphasizing their commitment to their treatment and the importance of following healthcare professionals' instructions for their recovery. This commitment was clear during their hospitalization and after leaving the hospital.

Patients recognized the significance of their active participation in the recovery process and expressed their willingness to follow medical instructions. For example, “I think I understood that I had to help them to help me, and then I had to obey, [that's why] I was there… if they tell me to stand upside down, I would do it.". Another patient emphasized the importance of patient involvement, “since it also depended on the encouragement degree, consuming food, maintaining the position that improved in terms of oxygenation”.

Some patients described the challenges they faced in adhering to the treatment but expressed their determination to carry it out, nonetheless. Patient 1 recounted an instance where they didn't feel like eating but recognized the necessity of doing so. They acknowledged the struggle to maintain their usual strength while being sick and expressed the importance of pushing oneself to overcome such challenges.

Furthermore, autonomy served as a source of motivation and strength for the participants when they left the hospital. Another patient shared their sense of empowerment: “from the moment I left the hospital, I was on my own. I was the one who cleaned myself… thanks God. I left the hospital still a little tired, but not to the degree that I had someone moving me… thank God I was able to fend for myself”. This self-reliance after leaving the hospital was seen as a positive outcome and a testament to their autonomy.

After being hospitalized, patients described how they continued to take responsibility for their own health. Patient 2, for instance, expressed the understanding that the treatment needed to be continued even after leaving the hospital, “aware that we still had to continue the treatment [did Dany follow it?] Of course… as they told me, we did it”. The hospitalization experience had a lasting impact on their commitment to self-care. Patient 6 reflected on their changed perspective on life, recognizing the value of the air they breathe and expressing a strong desire to avoid returning to the hospital: “If I don't take care of myself, I'm going to go back to the hospital. I, how do you say? Touch on wood. To tell the truth, I don't want to return there. They treated me very well, they treated me very well, but no thanks”.

In addition to autonomy, patients highlighted the importance of having a cheerful outlook and a will to live as contributing factors to their improvement. A patient recalled telling their family “I'm not going to die. Save those tears for me when you know I'm going to die, but I'm not going to die! I'm going to get out of here" (1-b,12). Patient 3 also emphasized the significance of inner strength, “I think it was very important to have the strength not to let oneself fall”.

Furthermore, patient 14 shared their initial confidence upon arriving at the hospital, “I told her not to worry, that I was happy, and she asked me why I was happy and I told her that because if I had COVID I was already in the hospital to be treated”. Their positive outlook proved their trust in receiving treatment and their willingness to confront the situation head-on. Overall, the patients showed a sense of personal responsibility for their own well-being. They acknowledged the need to continue their treatment outside the hospital, embraced a positive attitude, and displayed determination to overcome their illness and avoid readmission.

#### Informed consent

Within the dimension of autonomy, the patients discussed the concept of informed consent, highlighting two key factors: the right to information and freedom of choice. However, it is important to note that not all patients were able to personally sign the consent forms, and in some cases, a relative signed on their behalf.

Some patients expressed their understanding of the treatment and the details of their care, acknowledging that their doubts were clarified by the medical staff. Patient 7, for example, stated that all their doubts were addressed by doctors and nurses, “I was very restless and if I had doubts, I asked, and my doctors and nurses answered me, I did not have any doubts”. On the other hand, there were patients who did not have a clear recollection of the informed consent process. Patient 4 mentioned that their niece may have signed the consent form on their behalf, but they were not aware of the specifics: “they just admitted me, I don't know if she, my niece, signed any paper”. Patient 6 expressed uncertainty about whether the possibility of intubation was explained to them or their sister: “no they never told me, they didn't […] I don't know if they told her [sister] that she was going to be intubated, please a second,… not, they didn't tell her either”.

On the other hand, patients who were physically able had the opportunity to sign the informed consent themselves. Patient 5, for instance, mentioned signing the consent form but explicitly declined the option of being intubated. They expressed gratitude for being respected and supported in their decision by the medical staff. Patient 7 stated, “I was the one who gave my authorization to do whatever they wanted to me. Well of course if I wanted to be cured… I have small children, I think they also told my family what they did to me, but I was the one who said yes to everything”. In these cases, the patients felt empowered to make decisions about their treatment and appreciated the respect given to their choices.

Some patients reported feeling pressured by doctors to sign a consent form allowing them to be intubated, even when they initially refused the procedure. Patient 12 mentioned “Er, the doctors behaved very well except for one who was very insistent that I should sign a form to be intubated and I told him no”. Patient 13 also experienced similar pressure and felt uncomfortable with the insistence: “They kind of insisted that they wanted to intubate me. Maybe they were doing their job too, right? But I felt pretty bad about their insisting so much when I had already refused.” Both patients recognized the importance of having the freedom to make their own choices and were aware of their preferences.

These accounts show the importance of respecting patients' autonomy and freedom of choice in the informed consent process. While some patients met pressure to consent to certain procedures, others appreciated the opportunity to actively take part in the decision-making of their treatment.

### Health staff-patient relationship

Most of the patients expressed positive experiences about the care they received from doctors and nurses. Patient 1 mentioned having been treated diligently: "when I had to have a check-up, I don't know what they did to me, I took the stretcher bearer, it looked like I was going to receive an inheritance!”. Patient 5 expressed satisfaction with the treatment and recalled a staff member using endearing language while administering medicine. Patient 2 mentioned the kind and attentive behavior of the doctors: "normally 2 or 3 doctors went, and they were very kind to us, they went and asked how we felt, and I did see how I was evolving…". Patient 14 also highlighted the overall good treatment received from both doctors and nurses.

However, it is important to note that there were some patients who had negative experiences during their hospital stay. Patient 4 mentioned not receiving clear communication about their assigned healthcare professionals: “A doctor never came to tell me I'm your doctor […]”. They also recall “a very grumpy doctor who told [him he] had been seriously ill, that [he] was not going to leave and that [he] had to spend 20 days in the hospital”. Patient 6 shared a concerning encounter with a nurse who displayed a lack of empathy: “if you don't allow me to do my job, I'm going to intubate you”. Another patient mentioned the importance of kindness and good manners from healthcare professionals, noting that while not everything was bad during their hospitalization, they observed a lack of kindness, wishing for “maybe just a smile or the fact of treating you with kindness, as a sick person it makes you feel good […]”. These negative experiences highlight areas where improvements can be made in terms of communication, empathy, and patient-centered care.

### Illness

The patients shared their understanding of the disease, its development, and the treatment they received. Patient 2 mentioned: “[…] well, they did explain to us, they did the test and they told me that it was COVID and that this implied that it affects the lungs, that it can also affect the kidneys, the heart and this, obviously, the saturation that was very low”. Patient 6 talked about their initial misdiagnosis of pneumonia, which was later corrected to COVID-19. Others spoke of the sequelae of the disease, such as patient 4 who attests: "[…] I have been taking medication until today, right now a bit of shortness of breath and fatigue, just don’t disappear".

Regarding the treatment, patient 9 mentioned that “some medications were good, ceftriaxone and the one that was put in the shoulder and stomach for anticoagulant. But let's talk about pectin… what was it called? Ivermectin? something like that. Those were extraordinarily strong; All of this in the long run […] ended with my gut flora […]”. Patient 14 acknowledged being hospitalized early in the pandemic when knowledge about COVID-19 was limited. They expressed gratitude to the doctors for supplying the treatment they thought necessary and credited both the medical team and divine intervention for their recovery.

These patient experiences show varying levels of understanding of the disease, ranging from initial lack of knowledge to gaining insights during hospitalization. The patients also highlighted the impact of the disease on their health and the treatment efforts made by healthcare personnel, while also mentioning potential side effects or concerns associated with certain medications.

### The role of the family in the hospitalization of patients

The participants emphasized the vital role of family support and beliefs in their recovery process. The family served as a source of motivation, encouragement, and strength for the patients. Patient 1 expressed how the thought of her father waiting for her and the knowledge that her family was worried about her gave her a sense of shelter and determination to recover, remembering her father’s words: “daughter, courage, we are waiting for you here”. The hospital eased communication between patients and their families through videos, which provided great encouragement and stimulation. Patient 6 mentioned how watching the videos made by family members, hearing their supportive messages, and feeling the presence of loved ones played a significant role in maintaining the will to live.

Even in the isolating moments of being away from their families, thoughts of them served as a source of motivation. Patient 14 described how they “looked through the windows at the hills that can be seen in the west and thought: over there is my house; my family is there, so if I don't go out, I'm already close to them”. The patients also highlighted the importance of setting an example for their children and the impact it had on their recovery. Patient 1 mentioned staying strong and not breaking down for the sake of her daughters, while patient 5 mentioned that their family was responsible for obtaining the necessary medications and products as requested by the healthcare personnel.

Financial concerns and the burden of debts were also mentioned by patients as a source of worry for their families during their illness. Patient 12 expressed concern of how “all the debts increased, [he] hadn't worked and now [they] owe money, right? What was spent and all, that was [his] concern”. On the other hand, beliefs, particularly faith in God, played a significant role in providing hope and strength to the patients. Patient 6 referred to their illness as a test from God and found solace in their faith. Patient 15 shared that the fear of leaving their family behind and the love they had for God were motivating factors in their recovery.

In general, the patients recognized the vital support provided by their families and the influence of their beliefs in their journey to recovery. Family encouragement, material support, and the belief in a higher power served as sources of motivation, resilience, and hope throughout their hospitalization and beyond.

## Discussion

Living by the principles of bioethics is essential for physicians and residents as it helps the physician–patient relationship. The COVID-19 pandemic brought difficulties in communication and end-of-life care due to the lack of accurate prognoses and high threat to life. In these scenario problems in assertive communication can lead to misunderstandings and a sense of poor care from the patient's perspective. Research shows that disclosure of prognosis is not associated with emotional distress or harm to the physician–patient relationship but is associated with more advance care planning and comfort-oriented care [15].

The bioethics principles have become a widely used tool for ethical analysis. An expanded version of these principles looks to uphold the ethical standards that have been universally accepted in clinical practice. Our analysis has shown that these principles are based on the good habits of healthcare professionals. While adhering to ethical principles falls under the realm of deontological ethics, it is the ethics of virtue that provides the foundation that supports these principles [4]. Therefore, it is important to acknowledge the role that both deontological ethics and virtue ethics play in upholding the principles of bioethics.

In the healthcare system the person should be the focus, and a professional relationship with the health workers must be established to respect their rights, autonomy, and beliefs [15]. Trust is key for patients to acknowledge the complexity of medical services. A focus on the relationship can be seen as a strategic tool, among other things, which increases the success of the service [16]. The foundations of the dyadic relationship between physician and patient include benevolence, trust, and reciprocity. Research shows that patients develop trust when doctors treat them with empathy and transparency, sharing information and power [16]. Complaints are often related to ineffective communication, not the physician's skill set. Quality and satisfaction should be based on the perception of both the physician and the patient, such as perceived trust, experience, and personal views of the outcome. Shared decision-making can help patients regain control of their healthcare and better understand treatments and risks. Healthcare workers should also assess patients' feelings and emotions to provide better care. Building trust and patient communication can significantly improve customer satisfaction and the quality of medical services. A study on patient satisfaction shows that they value qualities like listening, empathy, communication, and interpersonal skills [16].

The patient-provider relationship directly affects patient care, and physicians' attitudes and behaviors change patients' facilitation of information, allowing for a more participative relationship [17]. Medical centers should aim to foster better provider attitudes and behaviors. The pandemic had a significant impact on patient experience. Some of the negative feeling related to longer wait times, challenges communicating with healthcare providers and feeling less engaged and connected to their healthcare providers, which may have contributed to a decline in patient satisfaction [18]. The lack of communication has had a direct correlation with decreased patient autonomy and a below-average symptom assessment [18].

In a study with COVID19 patients 5 primary themes were identified related to patient experience: gratitude, inconsistent communication, variable patient education, quality of patient care, and wait-times [18]. A different study also reported that patient satisfaction declined due to increased wait times, limited access to family members, and reduced communication with healthcare providers due to personal protective equipment (PPE) requirements [19]. However, patients appreciated healthcare providers who took the time to explain their medical condition, listened to their concerns, and treated them with respect. Patients seemed to express an abundance of gratitude towards the hospital staff for the quality of care and compassion during their hospital stay. Some of the hospitals are prioritizing communication and the use of technology to better engage with patients. This can be achieved by supplying clear and concise information, actively listening to patients, and addressing their concerns promptly [19].

The present research supplies insights into how the principles of bioethics are experienced through a qualitative study. The principles have become commonplace in ethical analysis, and an expanded version aims to rescue the universally accepted principles of clinical ethics [4]. The study reveals that these principles are supported by the good habits of healthcare professionals. It also reflects on the virtues and values that can shape the excellence of human character, such as altruism, generosity, and kindness. However, the question of whether habits, principles, or compliance with laws such as patients' and healthcare professionals' rights come first. In this case, it is to note that despite the difficulties faced by healthcare professionals, patients in a public hospital setting generally felt well cared for. This highlights the importance of the principles of bioethics in guiding ethical analysis while recognizing the limitations of these principles and the significance of virtue ethics in reflecting the true nature of human beings [4].

## Conclusions

The study highlights the strong correlation between bioethical principles and the relationships between healthcare professionals and patients. The patients in the study reported experiencing excellent care characterized by professionalism, compassion, fairness, and respect for their autonomy. These principles of bioethics, such as justice, charity, and autonomy, played a significant role in their positive experiences and contributed to their recovery.

The patients valued being treated with dignity and emphasized the importance of healthcare professionals respecting their individual values and rights. They appreciated the human aspect of care and the emphasis on treating each person with respect, regardless of their background or circumstances. However, it is acknowledged that there were instances where patient vulnerability was compromised, showing the need for ongoing attention to this issue and the continuous improvement of healthcare practices.

While bioethical principles supply a guiding framework, it is recognized that they alone may not be sufficient for a comprehensive ethical analysis. The ethics of virtue, which focus on cultivating virtues such as dignity, integrity, vulnerability, justice, beneficence, and autonomy, better capture how healthcare professionals manifest themselves in their roles. By embodying these virtues, healthcare professionals can supply more holistic and compassionate care to their patients.

It is important to recognize the dedication and sacrifices made by healthcare professionals, who willingly put their lives at risk to care for seriously ill patients during a time of uncertainty and limited treatment options. The pursuit of virtue, which requires discipline, commitment, and self-reflection, is a lifelong journey for individuals in the healthcare profession. By cultivating habits of virtue, healthcare professionals can not only supply better care but also contribute to society and lead more fulfilling lives.

### Supplementary Information

**Additional file 1: Appendix 1.** Interview guide.

## Data Availability

The datasets generated and analyzed during the current study are not publicly available due to confidentiality concerns but it are available from the corresponding author on reasonable request.
