# Supplementary material for: Perceptions of COVID-19 patients in the use of bioethical principles and the physician-patient relationship: a qualitative approach
Source: BMC Med Ethics. 2024 Feb 9;25:16. doi: 10.1186/s12910-024-01009-z (PMC10858506; doi:10.1186/s12910-024-01009-z)
Supplement: Supplementary file 1 — Additional file 1: Appendix 1. Interview guide. [file 12910_2024_1009_MOESM1_ESM.docx]

**Appendix 1.** *Interview guide.*

| DIGNITY |
| --- |
| For you, what does the dignity of a person consist of? |
| Do you think that all human beings have dignity? Why? |
| I could give examples of what it would be like not to treat a person with dignity… |
| In the case of a patient in general, for you, what would it be like to be treated with dignity by health personnel (doctor, nurse, social work, psychology)? |
| How would the dignity of a patient be violated by health personnel? |
| Did you receive a dignity treatment while you were sick with COVID19? Why? |
| CHARITY |
| Do you think that the procedures and medications received during your hospitalization were for your cure, for your own good? Why? |
| Do you have the impression that there was mistreatment during your illness that could have been avoided? Which one? |
| Did the health personnel explain your COVID 19 condition and the treatment you were receiving? Did you clarify your doubts? |
| JUSTICE |
| Do you consider that the necessary resources were used for your cure during your internment? Why? |
| Is there something you think you should have received that you were not given? How would it be? |
| Was the care received during the hospitalization for your condition good? |
| VULNERABILIDAD |
| Did COVID 19 make you feel fragile that put your existence at risk? Explain |
| Did the health personnel give you the security and confidence that they were doing everything possible for your recovery? |
| Do you think that you cooperated with the health personnel by being a good patient? Give examples |
| Did you feel fragile in the face of the need to receive help from the health personnel? What factors contributed to your feeling this fragility? |
| What did you learn (how did you feel) from having depended on the health personnel? |
| Did you feel fragile in the face of the need to receive help from your family? What factors contributed for you to feel this fragility? |
| What did you learn (how did you feel) from depending on your family? |
| What is the most important feeling you had when you were hospitalized? (well-being/discomfort) |
| What is the most important feeling you had when you were discharged? |
| AUTONOMY |
| Did your health conditions allow you to give your consent for your hospitalization or were your relatives the ones in charge?  Were you or your family explained about what informed consent is? |
| Did they explain to you or your family what it meant to be "intubated"? |
| OTHERS |
| How do you take care of your integrity? |
| Do you consider that taking care of your health is only the doctors’ and nurses’ responsibility? |
| What behaviors of a person can threaten their physical or moral integrity? Give examples. |
